# 

**Published:** 1870-04

**Authors:** 


### CONTENTS:

### ORIGINAL COMMUNICATIONS.

ARTICLE I.—Hyperæmia and Inflammatory Exudations of the Mucous Membrane of the Mouth. By A. German, D.D.S. .......... 557

ARTICLE II.—A Novel Case and Treatment. By Dr. S. J. Cobb, .......... 566

ARTICLE III.—Effects and Treatmentment of Salivary and Mucous Deposits. By Louis Augspath, D.D.S .......... 567

ARTICLE IV.—Disease of the Antrum, .......... 573

### SELECTED ARTICLES.

ARTICLE V.—Heavy Foils. By Henry S. Chase, M.D., D.D.S .......... 574

ARTICLE VI.—Mercury in India Rubber Dental Plates. By G. H. Perine, D.D.S .......... 576

ARTICLE VII.—Two Cases of Convulsions During Dentition Arrested by Sacrification of the Gums. By G. Stevenson Smith, L R.C.S.E .......... 579

ARTICTE VIII.—Interdental Splints for Fractures of Inferior Maxilla. By Dr. Geo. L. Fitch, .......... 583

ARTICLE IX.—The Inter-dependence of Diseases of the Teeth and of the Female Pelvic Organs. By Dr. N. W. Hawes, .......... 585

ARTICLE X.—Morgan's Plastic Gold, .......... 589

### MONTHLY SUMMARY.

Carbolic Acid, .......... 591

A Tooth Driven into the Antrum, .......... 592

Neuralgia and its Treatment by Electrization, .......... 593

Indication of Longevity, .......... 594

Green Line on the Gum from Copper Poisoning, .......... 594

Deaths from Chloroform, .......... 595

Blackened Teeth from Tea, .......... 595

Chloroform in Infantile Convulsions, .......... 595

### BIBLIOGRAPHICHAL NOTICES.

The Cell Doctrine. Its History and Present State. By James Tyson, M.D., .......... 596

### EDITORIAL DEPARTMENT.

Special Dental Journals, .......... 596
Anæsthetic Inhalation, .......... 597
Weston's Metal, .......... 598
South Carolina State Dental Association, .......... 601

